# Scattering effect of the high-index dielectric nanospheres for high performance hydrogenated amorphous silicon thin-film solar cells

**DOI:** 10.1038/srep30503

**Published:** 2016-07-26

**Authors:** Zhenhai Yang, Pingqi Gao, Cheng Zhang, Xiaofeng Li, Jichun Ye

**Affiliations:** 1 Ningbo Institute of Material Technology and Engineering, Chinese Academy of Sciences, Ningbo 315201, China; 2 College of Physics, Optoelectronics and Energy & Collaborative Innovation Center of Suzhou Nano Science and Technology, Soochow University, Suzhou 215006, China

## Abstract

Dielectric nanosphere arrays are considered as promising light-trapping designs with the capability of transforming the freely propagated sunlight into guided modes. This kinds of designs are especially beneficial to the ultrathin hydrogenated amorphous silicon (a-Si:H) solar cells due to the advantages of using lossless material and easily scalable assembly. In this paper, we demonstrate numerically that the front-sided integration of high-index subwavelength titanium dioxide (TiO_2_) nanosphere arrays can significantly enhance the light absorption in 100 nm-thick a-Si:H thin films and thus the power conversion efficiencies (PCEs) of related solar cells. The main reason behind is firmly attributed to the strong scattering effect excited by TiO_2_ nanospheres in the whole waveband, which contributes to coupling the light into a-Si:H layer *via* two typical ways: 1) in the short-waveband, the forward scattering of TiO_2_ nanospheres excite the Mie resonance, which focuses the light into the surface of the a-Si:H layer and thus provides a leaky channel; 2) in the long-waveband, the transverse waveguided modes caused by powerful scattering effectively couple the light into almost the whole active layer. Moreover, the finite-element simulations demonstrate that photocurrent density (*J*_ph_) can be up to 15.01 mA/cm^2^, which is 48.76% higher than that of flat system.

Although conventional hydrogenated amorphous silicon (a-Si:H) thin-film solar cells (TFSCs) encountered resistance of continuous improvement in efficiency, a-Si:H solar cells (SCs) with a relative thinner intrinsic layer are still of great interest because of many inherent advantages such as lower cost, high throughput, reduced detrimental Staebler-Wronski degradation effect^1^, as well as the numerous derivative applications in semi-transparent SCs^2^, top-sided sub-cells for tandem SCs^3^, flexible SCs^4^, etc. By scaling down the thickness of the active layer, the carrier transport loss is significantly decreased, and thus contributing to a good performance of the internal quantum progress inside the SCs. However, as light absorption is usually proportional to the film thickness, the ultrathin absorber thus appeals for advanced light-trapping strategies. For ultrathin a-Si:H layer (for example, 100 nm or less), the commonly used light-trapping option of textured transparent conducting oxide (TCO) with depth around several hundred nanometers (wavelength-scale) may cause severe degradations in efficiency, due to the leakage problem relates to the unsatisfied coating of a-Si:H. Alternatively, the emerging light-trapping schemes without structuring the active material, like photonic crystals (PC)^5^,^6^,^7^, plasmonics^8^,^9^,^10^, whispering gallery modes (WGMs)^11^,^12^,^13^,^14^,^15^ have been proved to be efficient ways to substantially improve the light-harvesting efficiencies by coupling incident light into the underlying absorber layer.

WGMs that are excited in the periodically arranged dielectric nanospheres can be coupled into particular modes of the solar cell and significantly enhance its efficiency by increasing the fraction of incident light absorbed^16^. As mentioned by Grandidier *et al.*^11^, through the use of wavelength-scale resonant SiO_2_-dielectric nanospheres, the highest current density of *J*_ph_ = 14.14 mA/cm^2^ can be achieved on 100 nm-thick a-Si:H TFSCs, representing an enhancement of 15% compared to the flat reference together with a well-designed antireflection coating. The broadband enhancement was explained when considering the spheres as a textured antireflection coating. Besides, as indicated by Wang *et al*. that the conversion efficiency can be improved from 8.10% to 9.89%, by introducing GaP (refractive index: *n* > 3) scatterers on the top surface of the flat a-Si:H TFSCs^17^. The underlying enhancement was attributed to the antireflection coating based on the excitation of a Mie resonance and the coupling of incident light into the waveguide modes by excited optical resonance, which both can greatly increase the light path in the active layer. However, the contribution to the photocurrent density is not satisfactory enough and the enhanced mechanism is also not very clear. Based on a fundamental theory of light management for SCs, Brongersma *et al.* proposed that the mechanisms on outstanding light-trapping can be classified as follows: 1) Fabry–Perot (FP) standing-wave resonance caused by the confinement light between top surface and the back-reflector; 2) optical (Mie) resonance, which is hybridized with a guided resonance in the underlying active layer; 3) guided resonance results from the periodic grating that ensures phase-matched coupling of a normally incident plane wave to a waveguided mode of the active layer; 4) diffracted modes, which are usually excited under oblique angle^18^. Specific to the case of light-harvesting by assembling high-index dielectric nanospheres, a quantitative analysis and a more detailed cognition are eagerly needed.

In this letter, we propose a new design by employing TiO_2_ (high index, *n* ≈ 2.7) dielectric nanospheres arrays (subwavelength scale) on top of a 100 nm-thick a-Si:H layer. The detailed simulation results show that absorption efficiency is significantly improved over a broad spectral band, leading to the photocurrent density increased from 10.09 mA/cm^2^ (flat design) to 15.01 mA/cm^2^. The optimized TFSCs exhibit a light-conversion efficiency up to 10.53%, which is improved by 43.85% over the flat counterparts. More importantly, the underlying mechanism for excellent light-trapping is attributed to scattering effect of the TiO_2_ nanospheres, judging by the direct evidence of exactly well-matched spectrum in scattering and absorption in the whole waveband. Combinational effects of the forward Mie scattering in the short-wavelength and waveguide modes excited by strong omnidirectional scattering in the long-wavelength clearly contribute to coupling the light into active layer and thus effectively increase the optical-path. Therefore, our work not only presents a novel and efficient strategy to design high performance TFSCs without active-layer structuring, but also resolves and confirms the optical coupling mechanism on high-index dielectric nanospheres, imposing a certain significance to manage the light for SCs.

Figure 1(a,b) describe the schematic diagrams of the proposed a-Si:H TFSCs under a superstrate configuration, which is made up of a tetragonal array with arranged TiO_2_-nanospheres, an indium tin oxide layer (ITO, 80 nm), an ultrathin photoactive region (a-Si:H, 100 nm), an aluminum-doped zinc oxide layer (AZO, 130 nm) and a back silver reflector^11^,^19^. TiO_2_ nanospheres are packed by an 80 nm ITO antireflective layer, with the aims to achieve outstanding impedance-match effect and to form electrical contact. With the optical constants from Palik^20^, we perform three-dimensional (3D) electromagnetic simulations by solving the Maxwell’s equations based on finite-element method (FEM)^21^. Specially, the optical constants (including the refractive index *n* and extinction coefficient *k*) for each materials were plotted in the Fig. S1 (supplement information). The photocurrent density (*J*_ph_) by spectrally integrating the absorption efficiency (*P*_abs_) (weighted by AM 1.5 G) is used to evaluate optical performance of the SCs^22^.

To identify the optimal design, a complete screening of the featured parameters including the lattice constant (*P*) and the diameter (*D*) of TiO_2_ nanospheres is carried out. Figure 1(c,d) plot the *J*_ph_ as a function of *P* (blue line) and *D* (red line), respectively. As reported previously, the subwavelength scale dielectric spheres can readily excite the resonance modes, so we tentatively fix *D* = 200 nm when sweeping *P* from 360 to 900 nm^23^. It is shown that *J*_ph_ tends to rise and then drop with a peak of 14.96 mA/cm^2^ at *P* = 510 nm. We then vary *D* from 0 to 600 nm with the same space of adjacent TiO_2_ nanospheres (i.e., *P* − *D* = 310 nm). Specially, as *D* = 0 nm, the TiO_2_ nanospheres are not presented at the front surface of SCs, so that it degrades to the conventionally flat system. With the increase of *D*, *J*_ph_ is substantially improved, especially when *D* > 150 nm. The maximum *J*_ph_ of 15.01 mA/cm^2^ occurs at *D* = 210 nm, with an enhancement of 48.76% compared to 10.09 mA/cm^2^ of flat reference (planar a-Si:H coated with 80 nm ITO). The improvement ratio of *J*_ph_ (48.76%) in our device is far beyond than that of wavelength-scale dielectric nanospheres design (WGMs) one (15%) in ref. 11. Here, we need to point out that the thickness of AZO and ITO is not the optimal design for both the flat and nanosphered TFSCs. The two coating layers were defined exactly same as ref. 11, a classical demonstration on WGM and a good reference to our design, for direct comparison purpose. The 3D plot of calculated photocurrent density (*J*_ph_) as a function of the thickness of AZO and ITO are shown in the Fig. S2 (supplement information).

Above photocurrent density values validate that strong optical enhancement can be achieved by properly setting parameters in the proposed device. We now examine the absorption spectral responses of the flat and proposed design in Fig. 2(a), where *D* = 210 nm and *P* = 510 nm for TiO_2_-nanosphered configuration are considered. Besides, the nanospheres design without ITO coating is also plotted as a comparison. As shown in Fig. 2(a), the optical absorption efficiency of the TiO_2_-nanosphered design with ITO coating is improved for *λ* > 370 nm, showing an almost full-spectral enhancement compared to the flat and solely TiO_2_-nanosphered design (without ITO coating). Obviously, the presence of the ITO coating upon TiO_2_ nanospheres contributes to the suppression of the reflection in the short-waveband and increase of the optical path in the long-waveband. The results demonstrate that the ITO coating plays a crucial role in coupling incident-light into SCs. The relatively weak optical response at *λ* < 370 nm, which is caused by strong parasitic absorption in TiO_2_ nanospheres and ITO layer, gives negligible contributions to the *J*_ph_ because the absorptions in this range are almost invalid to a-Si:H. However, attention should be paid on the long-wavelength range (*λ* > 550 nm), where the *P*_abs_ of flat a-Si:H TFSC is monotonously decreased due to the degenerative material-extinction coefficient, but the light-trapping capacity is dramatically improved by introducing TiO_2_ nanospheres arrays atop. For instance, at *λ* = 580 nm, *P*_abs_ = 41.67%, 67.66% and 80.45% for flat, TiO_2_ nanospheres with and without ITO coating systems, respectively. Especially, at the wavelengths close to the bandgap, taking *λ* = 720 nm for example, *P*_abs_ can be up to 40.34%, more than twenty times that of the flat (1.37%) and the design without ITO coating (1.76%) designs.

For correlation purpose, the scattering spectra of the TiO_2_ nanoshperes under the same configuration (i.e., *D* = 210 nm and *P* = 510 nm) is plotted in Fig. 2(b).In this simulation, the scattering efficiency (*Q*_sca_) is calculated by integrating energy flux densities under the entire surface area of nanosphere. Detailed information in this regard is presented in the formulas and Fig. S3 in the supplement information. As *λ* < 380 nm, the *Q*_sca_ is relatively low and smooth due to the parasitic loss of the TiO_2_ and ITO, which weaken the intensity of incident light and thus has negative effects on the scattering of TiO_2_ nanospheres. At 380 nm < *λ* < 540 nm, the dielectric TiO_2_ nanospheres together with the ITO layer act as a perfect antireflection layer, leading to slight increase in *Q*_sca_. This implies that the scattering efficiency is positively correlated with the absorption ability. While, as *λ* > 540 nm, *Q*_sca_ is significantly oscillated, benefiting from the secondary or repeated reflection light from the bottom reflector. The resonance wavelengths of the absorption peaks in the long-wavelengths range match well with that in the scattering one (see the vertical black-dotted lines in Fig. 2), which verifies that the improved light-trapping ability is closely related to the scattering effect of the TiO_2_ nanospheres.

For further insights into the electromagnetic coupling mechanism of the TiO_2_ nanosphered TFSC, the profiles of normalized electric field intensity inside the solar cells at representative wavelengths of 420 nm and 750 nm are shown in Fig. 3, in which the flat system is also given for comparison. The TiO_2_ nanospheres on the top surface behaves as a resonance nanocavity of approximate hexapolar symmetry in SC, giving rise to an improved absorption efficiency by exciting the Mie resonance at *λ* = 420 nm^16^,^24^,^25^. Compared to the flat system [Fig. 3(a)], the new design focuses the incident light into a region close to the bottom the TiO_2_ nanospheres, leading to a powerful energy spot [Fig. 3(b)]. The field distribution of the new design has a clear overlap with the a-Si:H layer, which implies that the light of radiative emission is effectively coupled into the TFSC by introducing a leaky channel for the light confined in the nanosphere, thus yielding a strong near-field electrical distribution and absorption efficiency in a-Si:H layer [can be seen in the Fig. 2(a)]. More clearly, the normalized integrated electric field and the calculated absorption at *λ* = 420 nm around the center of a TiO_2_ nanosphere are shown in Fig.3 (c) in cross-sectional manner. As shown in Fig. 3(c), the optical absorption of the a-Si:H layer concentrates on the front surface, while the parasitic absorption in TiO_2_ nanospheres and ITO layer is negligible. That means the forward scattering is dominant in all directions and thus TiO_2_ nanospheres in this wavelength can be thought as an optical lens that helps to focus the incident light into the active layer effectively.

As the wavelength increases, the absorption ability of a-Si:H is declined due to a low material-extinction coefficient, but the strong omnidirectional scattering ability that responsible for the outstanding light-trapping [in the Fig. 2(a,b)] is tremendously improved. Let us taking *λ* = 720 nm for an example, the effective optical absorption and increased light-trapping performance of the new design is mainly caused by coupling light into the active layer, so that the energy spots of the proposed design in the Fig. 3(e) spread almost all over the entire active layer than that of planar system [Fig. 3(d)]. The strong resonance coupling of light is explained by the powerful scattering of light from the resonant Mie scattering due to the high optical mode density of the high-index TiO_2_ nanospheres and a-Si:H substrate. Moreover, the resonant property leads to a large cross-sectional leaky channel and the guided resonance spread along the horizontal direction, so that even an array of TiO_2_ nanospheres only covers 41% (i.e., *D*/*P*) of the surface area nearly complete interaction with the incident-light can also be achieved. Unlike the absorption distribution in the Fig. 3(c), although the optical absorption in the ITO and Ag layer is increased [as the Fig. 3(f) shown], the absorption distribution still penetrates the whole active layer.

The outstanding light-trapping performance benefitted from the TiO_2_ nanospheres has been fully demonstrated in above sections. Therefore, it is necessary to examine the optical responses of the TFSCs and the scattering effect under various refractive indices of the dielectric nanospheres. Here, we don’t consider the parasitic absorption in the dielectric nanospheres (i.e., the extinction coefficient *k* = 0). Figure 4(a,c) display the light absorption (*P*_abs_) in the a-Si:H layer and normalized scattering efficiency (*Q*_sca_) of the TiO_2_ nanospheres as functions of the response wavelengths (*λ*) and the refractive indices (*n*), respectively. Besides, the relation of *J*_ph_ versus *n* is inserted in the Fig. 4(b). One can find that *J*_ph_ shows an unnoticeable change as *n* < 2, but a strong enhancement when *n* > 2. Moreover, as *n* > 2.8, *J*_ph_ is substantially improved (more than 15.0 mA/cm^2^). With the increase of *n*, the spheres can couple more Mie resonance modes, the intensity of *Q*_sca_ is strengthened and the response wavelengths is shifted to long-wavelength. In order to combine the theory obtained with practice, we further consider four typical dielectric nanospheres with refractive indices (*n*) of 1.5 (close to SiO_2_), 2.0 (close to SiN_x_), 2.7 (close to TiO_2_), and 3.5 (close to GaP), respectively. Figure 4(b,d) show the absorption and normalized scattering spectra of the optimized parameters (*D* = 210 nm and *P* = 510 nm). As shown in Fig. 4(b), for short-wavelength range (300 nm < *λ* < 600 nm), there is no obvious difference in the *P*_abs_. This means that *n* is not the decisive factor for texturing antireflection, and the perfect impedance-match condition can be fulfilled in a wide range of *n*. While, with increasing *n*, the light-harvesting ability of the TFSC is strengthened gradually at *λ* > 600 nm due to the improved scattering effect. Obviously, with *n* increases from 2.0 to 2.7, the *Q*_abs_ is significantly increased. As a result, *J*_ph_ of the TFSC is straightly enhanced (i.e., 12.02 mA/cm^2^ for *n* = 2.0, and 14.80 mA/cm^2^ for *n* = 2.7, respectively), as shown in the inset of Fig. 4(b). Therefore, the best light-trapping performance is determined by scattering efficiency when introducing the dielectric nanospheres atop, in which high-index is more advantageous.

Finally, based on the carrier drift-diffusion and Poisson’s equations, electrical evaluations of addressing the carrier transport and collection process are performed for the three systems mentioned above [in the Fig. 2(a)]. The detailed information on electrical simulation and electrical parameters (including carrier concentration, carrier mobility, lifetime, recombination coefficients, etc.) can be found in the supplement information as well as our previous publications^26^,^27^,^28^. Figure 5(a) exhibits the calculated external quantum efficiency (EQE) spectra of the TiO_2_ nanospheres with/without ITO coating and flat a-Si:H TFSCs, respectively. The EQE spectra of the three types of SCs show slightly lower than that of *P*_abs_ [as indicated in Fig. 2(a)] due to the carrier recombination loss. For the sample combined with TiO_2_ nanospheres and ITO, a broadband enhancement (*λ* > 370 nm) for EQE is achieved, contributing to a high short circuit current density (*J*_sc_). Figure 5(b) illustrates the current-voltage (*J*−*V*) characteristics of the three types of TFSCs, from which electrical parameters such as *J*_sc_, open-circuit voltage (*V*_oc_), fill factor (*FF*), and light-conversion efficiency (*η*) can be obtained. Apparently, *J*_sc_ of the proposed device with TiO_2_ nanospheres and ITO can be improved to 13.34 mA/cm^2^ with an enhancement ratio of 42.07% and 29.01% when separately comparing to the flat (*J*_sc_ = 9.39 mA/cm^2^) and the TiO_2_-nanosphered sample without ITO coating (*J*_sc_ = 10.34 mA/cm^2^) cases. Besides, the improved *J*_sc_ is responsible for the slightly increased *V*_oc_ (i.e., 903, 906 and 914 mV for the three designs). As a result, under an approximate *FF* (i.e., 86.32%, 86.35% and 86.36%), the TiO_2_-nanosphered TFSC with ITO coating achieves a higher *η* of 10.53%, with an enhancement ratio of 43.85% (30.16%), compared to 7.32% (8.09%) of the conventional flat with ITO coating (TiO_2_-nanosphered TFSCs without ITO coating) design. To have a well understanding of the electrical recombination and transport processes, we have presented the spatial distributions of the stabilized electrical parameters (including carrier generation, electron concentration, hole concentration, and bulk recombination in the Fig. S4 (supplement information). As a whole, the new design shows outstanding performance in both light-trapping and electrical transport process.

In summary, we present a novel approach to enhance the light-trapping capability and the light conversion efficiency of the ultrathin a-Si:H solar cells by introducing high-index TiO_2_ nanosphere arrays together with ITO coating on top of the a-Si:H layer. The simulation results show that absorption efficiency is significantly improved over a broad spectral band, leading to the photocurrent density increased from 10.09 mA/cm^2^ (flat design) to 15.01 mA/cm^2^ in 100 nm-thick active layer of a-Si:H. The optimized thin cell exhibits a light-conversion efficiency up to 10.53%, representing a improvement of 43.85% over its flat counterpart. The underlying mechanism is well explained by the normalized scattering spectrum and the electric field profile associated with the absorption distributions. The Mie resonance as well as guided modes that excited by strong scattering effect afford leaky channels, so that light can be effectively couple into the active layer. This light-trapping design offers great flexibility in dielectric nanospheres with different indices and can be easily extended to other thin-film solar cells with versatile materials.

## Additional Information

**How to cite this article**: Yang, Z. *et al.* Scattering effect of the high-index dielectric nanospheres for high performance hydrogenated amorphous silicon thin-film solar cells. *Sci. Rep.* **6**, 30503; doi: 10.1038/srep30503 (2016).

## Supplementary Material

Supplementary Information

## Figures and Tables

**Figure 1:**
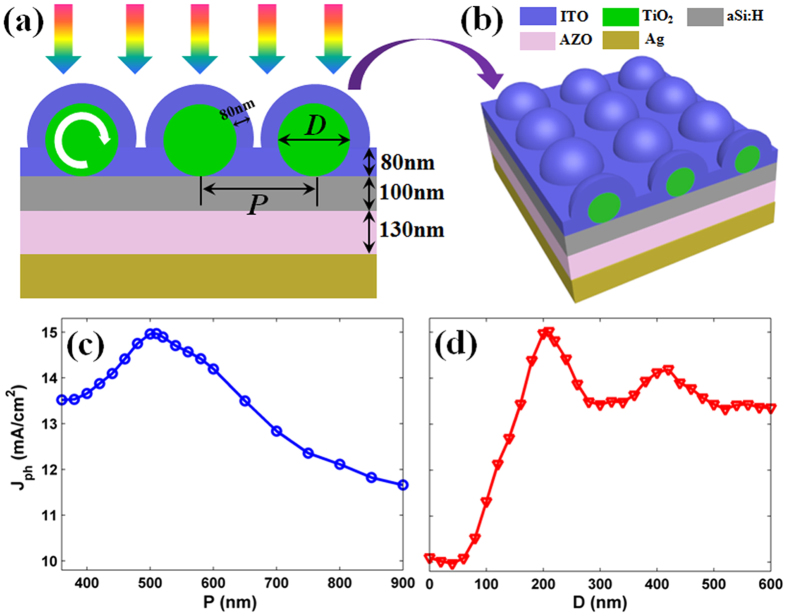
Schematic configuration and the tunability of optical resonance. Cross-sectional schematic diagram of TFSCs (**a**), and three-dimensional (3D) diagram (**b**) of the ultrathin a-Si:H solar cells with a tetragonal array of arranged TiO_2_-nanospheres, and ITO coating upon the front of the a-Si:H layer. Photocurrent density (*J*_ph_) versus the lattice constant: *P* (**c**) and the diameter of TiO_2_ nanospheres: *D* (**d**).

**Figure 2:**
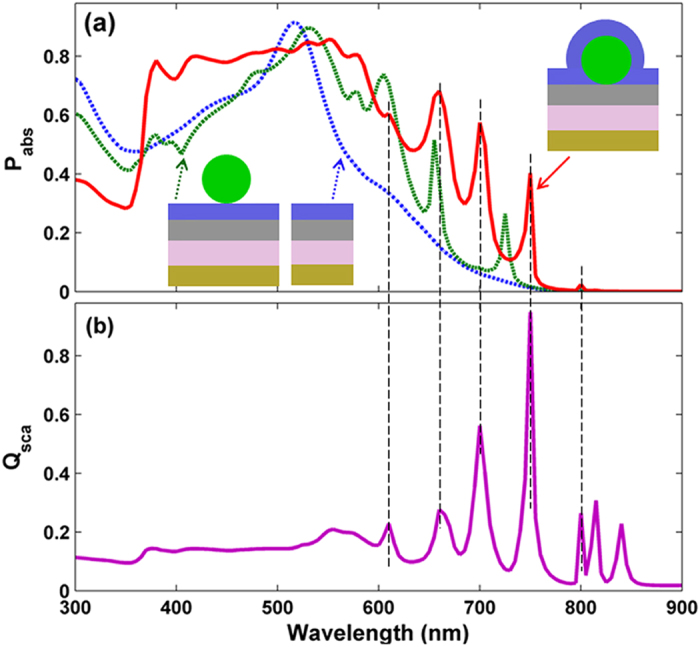
Absorption and scattering spectra. (**a**) Absorption spectra (*P*_abs_) under flat, TiO_2_-nanosphered without and with ITO coating designs, and (**b**) normalized scattering spectrum (*Q*_sca_) under TiO_2_-nanosphered with ITO coating system.

**Figure 3:**
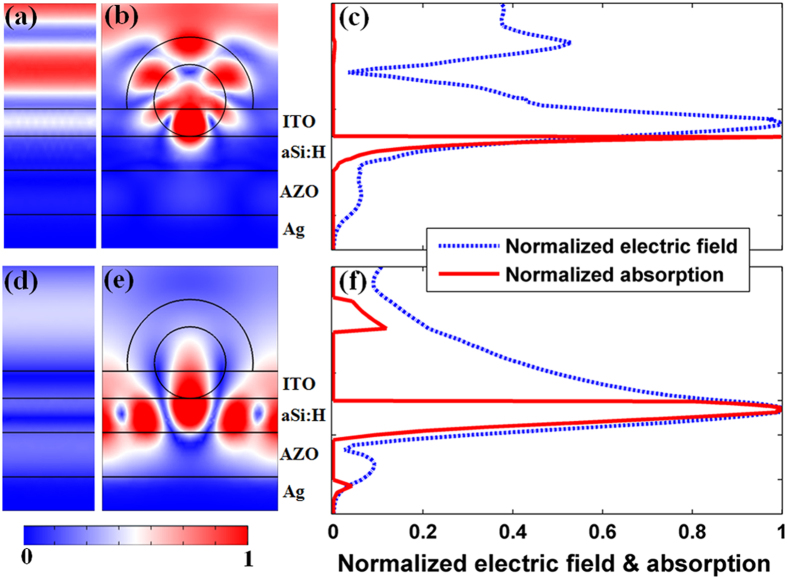
Electric field patterns, and electric field as well as absorption intensity versus position of the solar cells. Cross-sectional normalized electric field distributions inside the a-Si:H TFSCs at *λ* = 420 nm for (**a**,**b**) and *λ* = 750 nm for (**d**,**e**), (**c**,**f**) are integrated electric field and calculated absorption of a cross section at the center of the sphere for TiO_2_-nanosphered designs.

**Figure 4:**
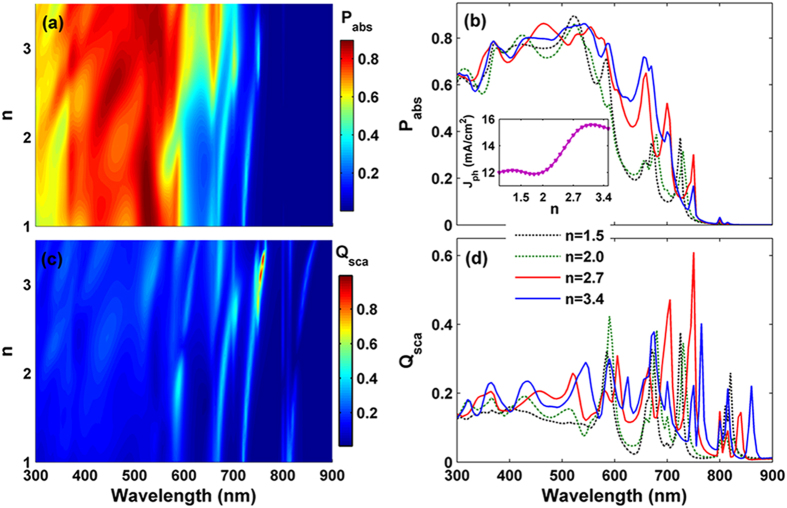
Absorption and scattering spectra versus refractive indices *n*. (**a**) *P*_abs_ and (**c**) *Q*_sca_ of TFSCs as functions of *λ* and *n*, (**b**) *P*_abs_ and (**d**) *Q*_sca_ spectrum under four typical refractive indices *n*. The photocurrent density (*J*_ph_) as a function of *n* is inserted in (**b**).

**Figure 5:**
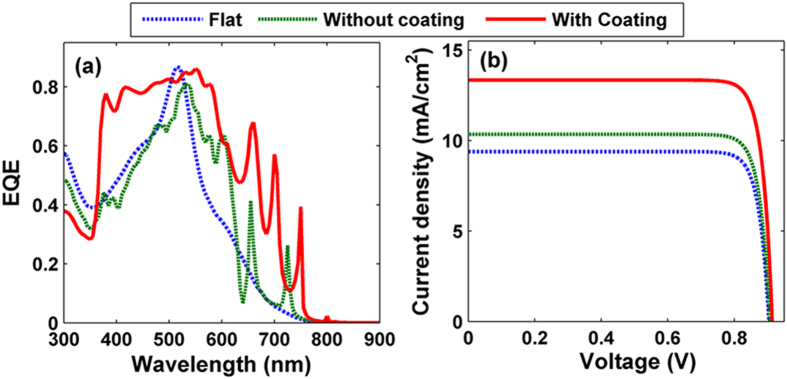
EQE and *I*-*V* characteristic curve. (**a**) EQE spectra, and (**b**) photocurrent as a function of the forward electrical bias *V* of TFSCs under flat, TiO_2_-nanosphered without and with ITO coating TFSCs.
