# Supplementary material for: Scattering effect of the high-index dielectric nanospheres for high performance hydrogenated amorphous silicon thin-film solar cells
Source: Sci Rep. 2016 Jul 26;6:30503. doi: 10.1038/srep30503 (PMC4960586; doi:10.1038/srep30503)
Supplement: Supplementary Information [file srep30503-s1.pdf]

Scattering effect of the high-index dielectric nanospheres for high performance hydrogenated amorphous silicon thin-film solar cells

Zhenhai Yang[1], Pingqi Gao[1,*], Cheng Zhang[2], Xiaofeng Li[2], and Jichun Ye[1,*]

[1]Ningbo Institute of Material Technology and Engineering, Chinese Academy of Sciences, Ningbo 315201, China

[2]College of Physics, Optoelectronics and Energy & Collaborative Innovation Center of Suzhou Nano Science and Technology, Soochow University, Suzhou 215006, China

Corresponding author: gaopingqi@nimte.ac.cn (PG); jichun.ye@nimte.ac.cn (JY)

Supplement information

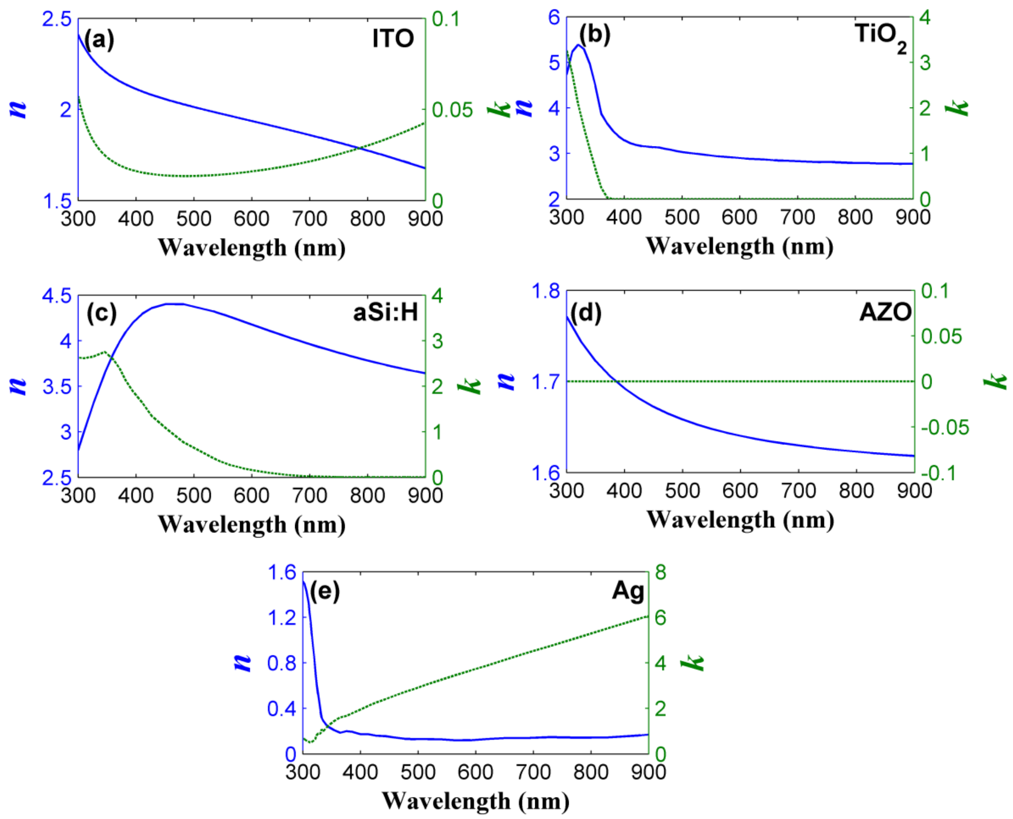

Fig. S1. The refractive index *n* and extinction coefficient *k* for (a) ITO, (b) TiO_2, (c) a-Si:H, (d) AZO, and (e) Ag, respectively.

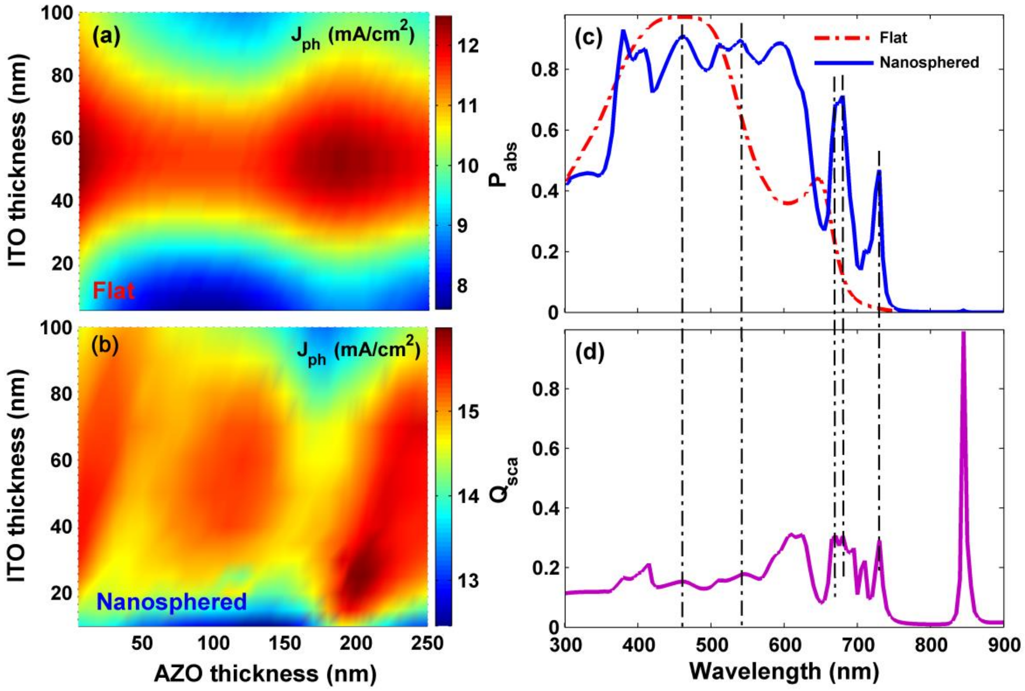

Fig. S2. (a)/(b) photocurrent density (J_{ph}) versus thickness of ITO and AZO for the flat/nanosphered TFSCs, (c) absorption spectra (P_{abs}) under flat and nanosphered designs, and (d) normalized scattering spectrum (Q_{sca}) under TiO_2-nanosphered system.

In this study, on the basic of electromagnetic field distributions, we calculated the scattering efficiency (Q_{sca}) by integrating energy flux densities under the entire surface area of nanosphere. In detail, it can be expressed as the following formulas:

$$Q_{\mathrm{sca}} = \oiint P_{\mathrm{sca}} \mathrm{ds} \tag{1}$$

$$P_{\mathrm{sca}} = n_{\mathrm{x}} \cdot P_{\mathrm{scax}} + n_{\mathrm{y}} \cdot P_{\mathrm{scay}} + n_{\mathrm{z}} \cdot P_{\mathrm{scaz}} \tag{2}$$

$$P_{\mathrm{scax}} = \frac{1}{2}\mathrm{Re}(\boldsymbol{E}_{\mathrm{y}} \times \boldsymbol{H}_{\mathrm{z}} - \boldsymbol{E}_{\mathrm{z}} \times \boldsymbol{H}_{\mathrm{y}}) \tag{3}$$

$$P_{\mathrm{scay}} = \frac{1}{2}\mathrm{Re}(\boldsymbol{E}_{\mathrm{z}} \times \boldsymbol{H}_{\mathrm{x}} - \boldsymbol{E}_{\mathrm{x}} \times \boldsymbol{H}_{\mathrm{z}}) \tag{4}$$

$$P_{\mathrm{scaz}} = \frac{1}{2}\mathrm{Re}(\boldsymbol{E}_{\mathrm{x}} \times \boldsymbol{H}_{\mathrm{y}} - \boldsymbol{E}_{\mathrm{y}} \times \boldsymbol{H}_{\mathrm{x}}) \tag{5}$$

where n_x, n_y and n_z are the refractive indexes, P_{scaz}, P_{scaz} and P_{scaz} are the energy flux densities, E_x, E_y and E_z (H_x, H_y and H_z) are the electric (magnetic) fields along x, y and z directions, respectively. S is the surface area of nanosphere. To well address this problem, we presented the P_{scaz} values with the space angle (in the yz plane) as an example (as shown in Fig. S3).

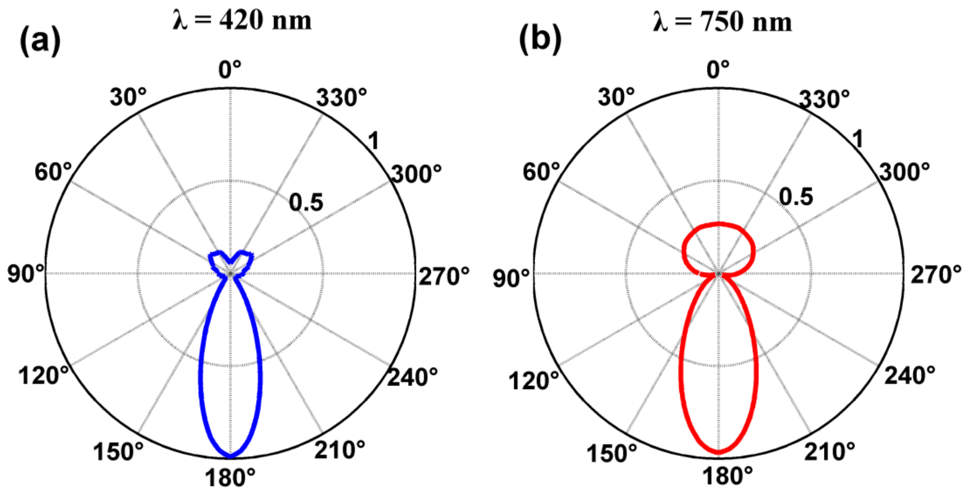

Fig. S3. P_{scaz} as a function of the angle under (a) λ = 420 nm, and (b) 750 nm, respectively.

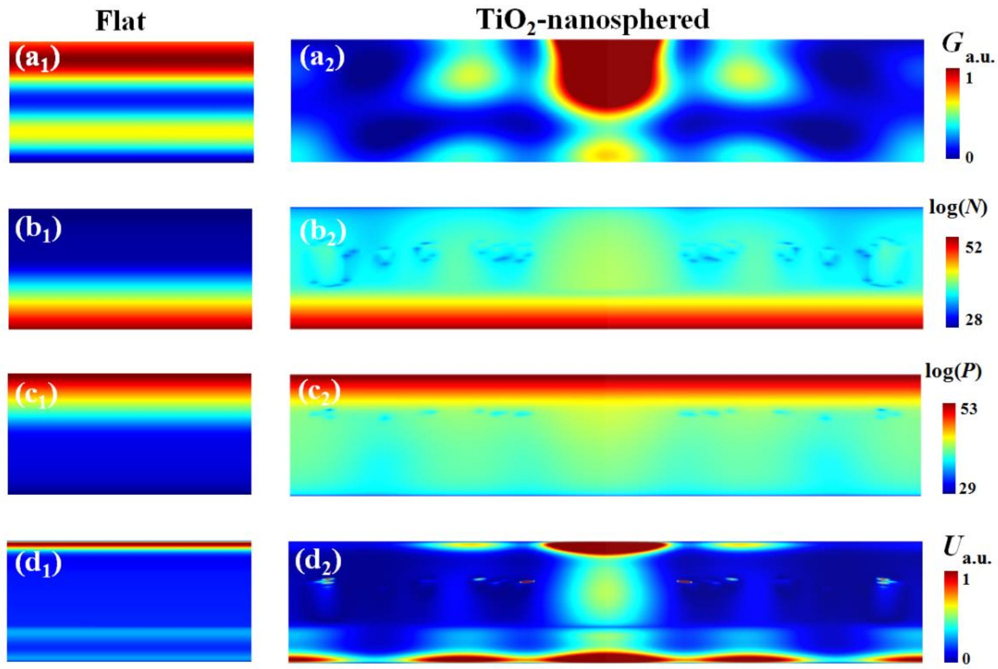

Fig. S4. The spatial distributions of the stabilized carrier generation (a_1)/(a_2), electron concentration (b_1)/(b_2), hole concentration (c_1)/(c_2), and bulk recombination (d_1)/(d_2) inside the a-Si:H layer at the wavelength of 500nm for flat/TiO_2 nanosphered TFSCs, respectively.

The three dimensional (3D) transport equations for electrons and holes in this study can be expressed as:

$$\nabla(-D_n \nabla n + n\mu_n \nabla \Phi) = G - U \tag{6}$$

$$\nabla\left(-D_p \nabla p - p\mu_p \nabla \Phi\right) = G - U \tag{7}$$

where n (p) is the electron (hole) concentration, $D_n = \mu_n K_B T/q$ ($D_p = \mu_p K_B T/q$) is the electron (hole) diffusion coefficient, μ_n (μ_p) is the electron (hole) mobility, K_B is Boltzmann's constant, T is the operating temperature, q is the electron charge, Φ is the electrostatic potential. The electrostatic potential Φ is determined by the charge profiles in the device according to the Poisson's equation:

$$\nabla^2\Phi = \frac{q}{\varepsilon}(n - p - N_d + N_a) \tag{8}$$

where ε is the material permittivity, N_d (N_a) is the concentration of the ionized donor (acceptor). The wavelength-dependent photocarrier generation rate $G(\lambda)$ can be expressed as following formula:

$$G(\lambda) = \frac{\varepsilon''(\lambda)|E(\lambda)|^2}{2\hbar}\Phi_{\mathrm{AM1.5}}(\lambda)\mathrm{d}\lambda \tag{9}$$

where ε'' is the imaginary part of the permittivity, E is the electric field, and $\hbar$ is the reduced Planck constant. U includes contributions from Shockley-Read-Hall, radiative, and Auger recombinations:

$$U_{\mathrm{bulk}} = U_{\mathrm{SRH}} + U_{\mathrm{Aug}} + U_{\mathrm{Rad}} \tag{10}$$

$$U_{\mathrm{SRH}} = \frac{np - n_i^2}{\tau_n(p+n_i)+\tau_p(n+n_i)} \tag{11}$$

$$U_{\mathrm{Aug}} = (C_n n + C_p p)(np - n_i^2) \tag{12}$$

$$U_{\mathrm{Rad}} = B_{rad}(np - n_i^2) \tag{13}$$

where τ_n (τ_p) is the electron (hole) lifetime, n_i is the intrinsic carrier concentration, B_{rad} is the coefficient of bimolecular radiative recombination, and C_n (C_p) the electron (hole) Auger coefficient. The short-current density (J_{sc}) is calculated by integrating the *EQE* spectrum of the cell under the standard AM1.5G illumination.

$$J_{\mathrm{sc}} = \int_{300nm}^{850nm}\frac{e\lambda}{hc}\Phi_{\mathrm{AM1.5}}(\lambda)EQE(\lambda)\mathrm{d}\lambda \tag{14}$$

where e is the unit charge, h the Plank constant, c the speed of light in vacuum, and $\Phi_{\mathrm{AM1.5}}$ the solar spectral irradiance under air mass 1.5.

Table. S1 Parameters used in this simulation

Variables	Values	Unit
N_a	1.3×10^{17}	cm^{-3}
N_d	4.3×10^{16}	cm^{-3}
T	300	K
τ_n	$1.7 - 2$	μs
τ_p	$0.34 - 2$	μs
μ_n	$4.6\times10^{-2} - 1$	cm^2/Vs
μ_n	$9.2\times10^{-3} - 0.5$	cm^2/Vs
ε	11.7	
C_n	1×10^{-30}	cm^6/s
C_p	1×10^{-30}	cm^6/s
B_{rad}	7.2×10^{-10}	cm^3/s
n_i	5×10^{7}	cm^{-3}
